# An innovative *in vitro* model for studying the biology of cardiac fibroblasts originating from the epicardium

**DOI:** 10.1242/dmm.052601

**Published:** 2026-04-02

**Authors:** Claudia Müller-Sánchez, María Gertrudis Muñiz-Banciella, Manuel Reina, Francesc X. Soriano, Ofelia M. Martínez-Estrada

**Affiliations:** ^1^ Celltec-UB, Department of Cell Biology, Physiology, and Immunology, Faculty of Biology, University of Barcelona, 08028 Barcelona, Spain; ^2^ Institute of Biomedicine (IBUB), University of Barcelona, 08028 Barcelona, Spain; ^3^ Institute of Neurosciences, University of Barcelona, 08035 Barcelona, Spain

**Keywords:** Cardiac fibroblasts, Epicardium, Wt1 transgenic mouse models

## Abstract

Embryonic epicardium is a major source of cardiac fibroblasts (CFs), which play essential roles in heart development and response to heart injury. In this study, we developed a novel mouse model to identify distinct populations of epicardium-derived CFs. Our Wt1^GFP/+^;Wt1Cre;ROSA26-tdRFP model enables lineage tracing of Wt1Cre-labeled (RFP^+^) fibroblasts and the identification of cells actively expressing WT1 (GFP^+^). Flow cytometry at early postnatal stages showed that RFP^+^ cells form a heterogeneous stromal population, with 20.13% co-expressing GFP, indicating persistent WT1 expression in a subset. We successfully immortalized RFP^+^ cardiac stromal cells, which are highly enriched in fibroblasts, by excluding other Wt1Cre-active cell types. Through culture condition optimization, we could selectively expand or differentiate specific fibroblast subpopulations, increasing the utility of the model. These immortalized cells, carrying an integrated WT1 reporter system, provide a robust *in vitro* platform to study fibroblast activation, differentiation and plasticity under defined conditions.

## Teaser

**Summary:** This study presents a novel *in vitro* model for investigating the activation, identity and functional properties of epicardium-derived cardiac fibroblasts.

## INTRODUCTION

Cardiac fibroblasts (CFs) are essential non-myocyte cells that play a crucial role in heart development and homeostasis (Tallquist, 2018). They are among the most abundant non-cardiomyocyte cell types in the postnatal heart and responsible for extensive extracellular matrix (ECM) deposition. Beyond their structural role, CFs produce paracrine factors critical to heart development, myocardial growth, functional maturation and blood vessel formation (Acharya et al., 2012; Hortells et al., 2020; Ieda et al., 2009). They are also central to the formation of pathological fibrosis, a hallmark of various cardiovascular diseases, by promoting excessive ECM deposition and tissue remodeling (Humeres and Frangogiannis, 2019).

The understanding of CF biology has advanced significantly over the past decade (Tallquist and Molkentin, 2017). Although several origins have been proposed, lineage tracing studies in mice have unequivocally demonstrated that adult resident CFs primarily arise from the embryonic epicardium and endocardium, with the epicardium being the predominant source (Moore-Morris et al., 2014; Tallquist, 2018). Notably, the population of resident CFs of epicardial origin expands in response to adult cardiac fibrosis, such as that induced by pressure overload, fibroelastosis or myocardial infarction (Forte et al., 2020; Moore-Morris et al., 2014; Ruiz-Villalba et al., 2015; Zhang et al., 2017).

Compared with other cell types, the absence of unique fibroblast markers, coupled with their inherent heterogeneity, has made the isolation of fibroblasts particularly challenging when using conventional techniques, such as fluorescence-activated cell sorting (FACS) (Tallquist and Molkentin, 2017). Various genetic mouse models have, therefore, been developed to facilitate fibroblast identification. Some models employ direct fibroblast reporter systems, such as the reporter cell lines Pdgfra-GFP or Col1a1-GFP that express Pdgfra or Col1a1, respectively, tagged to green fluorescent protein (GFP) (Hamilton et al., 2003; Yata et al., 2003). Additionally, Cre transgenic mouse models have significantly improved the identification and characterization of CFs (Acharya et al., 2011; Tallquist and Molkentin, 2017; Wessels et al., 2012; Zhou et al., 2008).

The *Wt1* gene encodes the WT1 transcription factor, a zinc finger protein essential for heart development (Hastie, 2017; Moore et al., 1999). WT1 is highly expressed in the epicardium and epicardial derived cells (EPDCs) during heart development, where it plays a critical role in their formation and function (Martínez-Estrada et al., 2010; Moore et al., 1999; Velecela et al., 2019). In addition to its epicardial roles, WT1 is also expressed in coronary endothelial cells, where it contributes to the development of the coronary vasculature (Cano et al., 2016; Duim et al., 2015; Ramiro-Pareta et al., 2023). Epicardial derivatives, such as CFs, also express WT1 both during heart development and following myocardial infarction (Braitsch and Yutzey, 2013; Pérez-Pomares et al., 2002). These expression patterns, initially identified through immunostaining, have been confirmed through recent transcriptomic analyses (Deng et al., 2025; Feng et al., 2022).

Over the past decades, a variety of transgenic mouse models based on WT1 expression has been developed (Hosen et al., 2007; Moore et al., 1998; Wessels et al., 2012; Wilm et al., 2005; Zhou et al., 2008). In addition to *Wt1* reporter mice with GFP knock-in (KI), both constitutive and inducible Wt1Cre models, generated by using KI or bacterial artificial chromosome approaches incorporating *Wt1* regulatory sequences, are widely used to study organ development, tissue repair and the lineage contributions of WT1^+^ cells (Hosen et al., 2007; Wessels et al., 2012; Zhou et al., 2008). Among these, one of the most commonly used tools is the constitutive BAC Wt1Cre transgenic line, which robustly labels epicardial cells and EPDCs, along with their progeny, including mural cells and CFs (Wessels et al., 2012). Although Cre activity is also observed in additional lineages – such as coronary endothelial and cardiomyocytes (Albendea-Gomez et al., 2025; Cano et al., 2016; Wessels et al., 2012) – the BAC Wt1Cre line remains a key model for studying resident fibroblasts of epicardial origin and has been widely used for this purpose in several studies (Forte et al., 2020; Moore-Morris et al., 2018; Ruiz-Villalba et al., 2015).

In this study, we combined the previously established constitutive BAC Wt1Cre transgenic mouse with a Wt1GFP KI model to generate a novel Wt1^GFP/+^; Wt1Cre; ROSA26-tdRFP mouse model, allowing simultaneous lineage tracing and visualization of WT1-expressing cells and their progeny (Hosen et al., 2007; Wessels et al., 2012). This includes WT1 lineage cells that are red fluorescent protein (RFP) positive (RFP^+^), while cells actively expressing WT1 are GFP positive (GFP^+^). We, subsequently, established immortalized CFs from this model, which represent a valuable and versatile *in vitro* tool for dissecting the molecular mechanisms that underlie fibroblast activation, differentiation and plasticity.

## RESULTS

### Characterizing cardiac cells from the Wt1^GFP/+^;Wt1Cre;ROSA26-tdRFP mouse model

Although several studies have used Wt1Cre mouse models to study fibroblast formation during cardiac development and disease states, none has combined lineage tracing with a *Wt1* reporter model to simultaneously identify both Wt1Cre progeny and Wt1-expressing cells, which can be used to FACS-isolate different populations of Wt1Cre^+^ cells by using FACS (Forte et al., 2020; Moore-Morris et al., 2014; Ruiz-Villalba et al., 2015; Wessels et al., 2012). We aimed to generate such a model by crossbreeding Wt1Cre;ROSA26-tdRFP mice with Wt1^GFP/+^ mice. We can use this strategy because, although the BAC Wt1Cre transgenic mouse model carries an IRES–GFP–Cre cassette downstream of the translation stop site of the *Wt1* gene, GFP expression remains undetectable for unknown reasons. This phenomenon has been reported since the model was first generated (Wessels et al., 2012).

In the Wt1^GFP/+^ model, GFP is knocked into exon 1 and driven by the endogenous Wt1 regulatory sequences, providing a GFP reporter that faithfully reflects *Wt1* expression (Hosen et al., 2007; Velecela et al., 2019). Although Wt1^GFP/+^ mice possess only a single functional *Wt1* allele, the potential effects of *Wt1* haploinsufficiency on the heart have not been formally assessed in this or any other heterozygous model (Martínez-Estrada et al., 2010; Velecela et al., 2019; Zhou et al., 2008). In addition, the copy number of the BAC Wt1Cre transgene – and, therefore, its possible contribution to total *Wt1* transcript levels – remains unknown; although we anticipate minimal impact, given that this Cre line generates *Wt1* knockout mice (Torres-Cano et al., 2022). To directly assess this issue, we quantified *Wt1* expression across all genotypes generated from the Wt1Cre;ROSA26-tdRFP×Wt1^GFP/+^ cross. Quantitative real-time PCR (qRT-PCR) analysis showed that *Wt1* transcript levels in all the genotypes, including triple-transgenic Wt1^GFP/+;^ Wt1Cre;ROSA26-tdRFP mice, were comparable to those of control littermates (Fig. S1). These results demonstrate that neither the BAC *Wt1Cre* transgene nor *Wt1* haploinsufficiency altered endogenous *Wt1* dosage in the early postnatal heart.

**Fig. 1.**
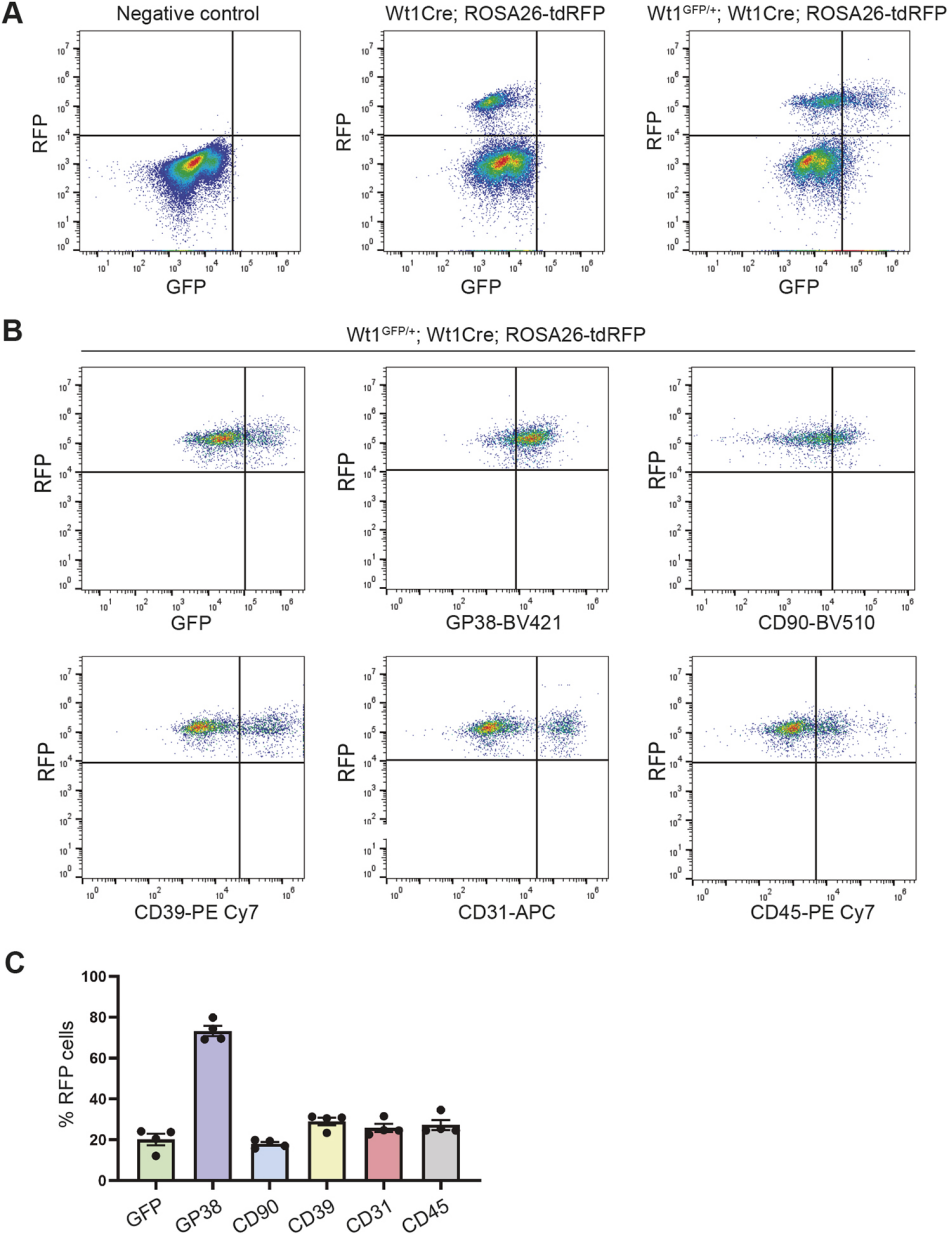
**Generation and characterization of freshly isolated cardiac cells from the Wt1^GFP/+^;Wt1Cre;ROSA26-tdRFP mouse model.** (A) Flow cytometry analysis of enzymatically digested heart ventricles obtained from Wt1^GFP/+^;Wt1Cre;ROSA26-tdRFP mice at postnatal day 2 reveals that a subset of RFP^+^ cells retains sustained GFP expression. Littermate negative mice were used to establish gating parameters. (B,C) Representative flow cytometry plots for identification of RFP^+^ subsets and corresponding quantification. Data are presented as mean±s.e.m. (*n*=4).

In the new model, Wt1^GFP/+^;Wt1Cre;ROSA26-tdRFP mice contain RFP^+^ cells that exhibit Cre recombinase activity, along with their progeny, while GFP^+^ cells express abundant WT1 levels. Flow cytometry analysis of ventricular cardiac cells at early postnatal stages showed that RFP^+^ cells represented 13.75% of the total cardiac cell population (Fig. S2). Of these, 20.13% were GFP^+^, indicating sustained WT1 expression in a cell subset (Fig. 1A,B, Tables S1, S2).

To further define the RFP^+^ population, we used a panel of several antibodies (Fig. S2). We found that 25.83% of RFP^+^ cells expressed CD31 and 27.25% expressed CD45, confirming the presence of endothelial and hematopoietic cells within the RFP^+^ population (Fig. 1B,C, Table S2) (Cano et al., 2016; Pogontke et al., 2022). Notably, 69.61% of the RFP^+^ CD45^+^ cells and 76.34% of the RFP^+^ CD31^+^ cells co-expressed GFP (Table S3).

We also included marker proteins commonly used to identify stromal cell populations, including GP38 (encoded by *Pdpn*), CD90 (encoded by *Thy1*) and CD39 (encoded by *Entpd1*), all of which have been used to detect different stromal cell populations, including CFs (Furtado et al., 2014; Ieda et al., 2009; Lu and Insel, 2013; Mahtab et al., 2008; Stellato et al., 2019). Among the RFP^+^ cells, 73.25% were GP38^+^, 18.03% were CD90^+^ and 29.03% were CD39^+^ (Fig. 1B,C, Table S2). Of note, the percentage of GFP^+^ cells varied among these populations, accounting for 28.91% of GP38^+^, 36.46% of CD90^+^ and 71.04% of CD39^+^ cells (Table S4).

Taken together, these results demonstrate that the Wt1^GFP/+^;Wt1Cre;ROSA26-tdRFP mouse model allows the identification of different populations of Wt1Cre^+^ interstitial cells based on GFP expression.

### Immortalization of cardiac cells in the Wt1^GFP/+^;Wt1Cre;ROSA26-tdRFP mouse model

**Fig. 2.**
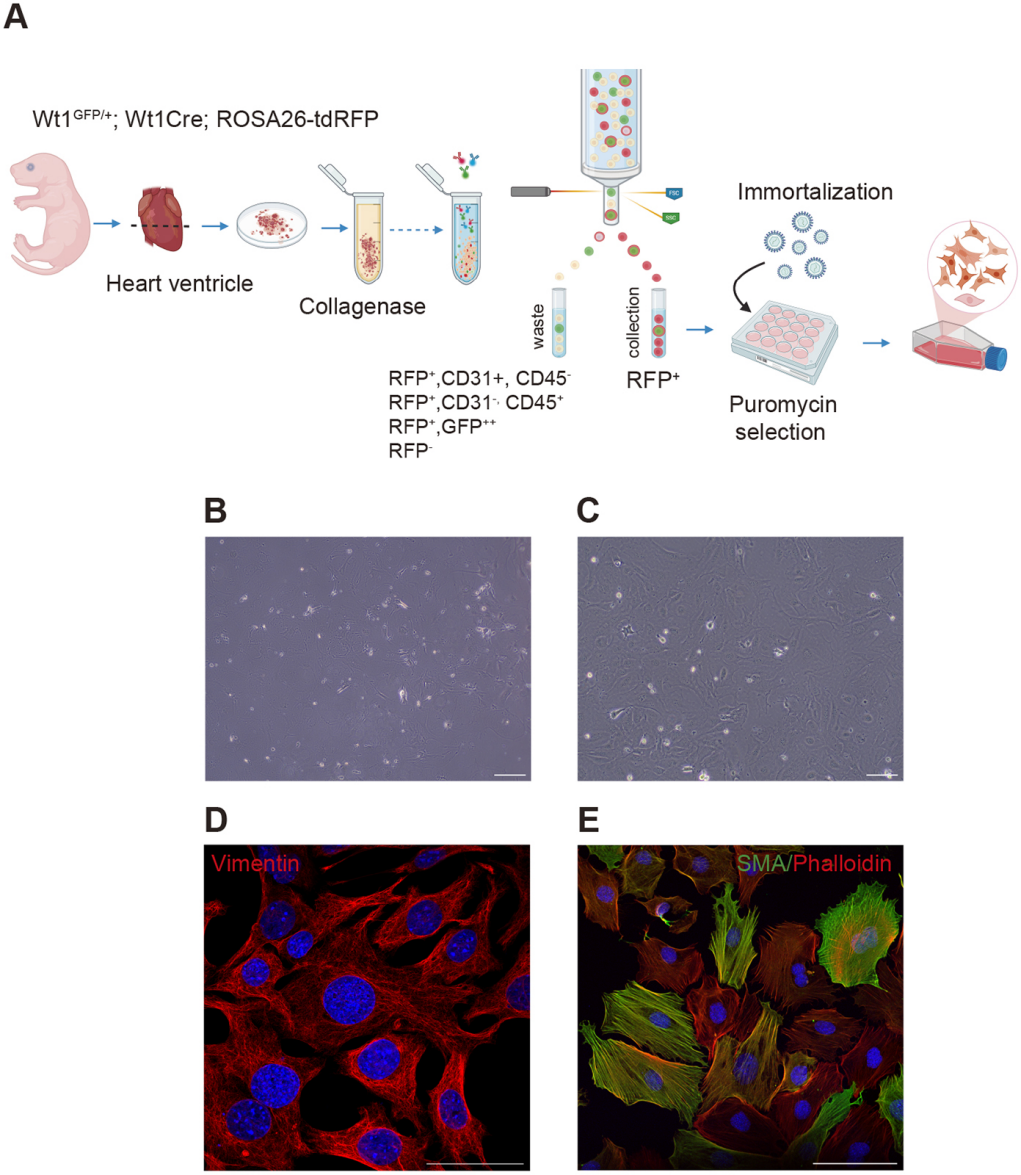
**Generation of immortalized cardiac cells from Wt1^GFP/+^;Wt1Cre;ROSA26-tdRFP mice.** (A) Workflow for the strategy used to generate and immortalize postnatal RFP^+^ cardiac stromal cells from Wt1^GFP/+^;Wt1Cre;ROSA26-tdRFP mice excluding endothelial cells (RFP^+^, CD31^+^, CD45^−^), hematopoietic cells (RFP^+^, CD31^−^, CD45^+^), and cells with the highest GFP expression (RFP^+^, GFP^++^). Created in BioRender by Martıńez-Estrada, O. M. (2025). https://BioRender.com/2iuf1eq. (B,C) Bright-field image of primary (B) and immortalized (C) RFP^+^ cardiac cells. (D) Immunofluorescence staining for the intermediate filament vimentin, demonstrating abundant vimentin expression in immortalized cells. (E) Confocal immunofluorescence staining for α-SMA (green) and phalloidin (red), exhibiting some patches of α-SMA^+^ cells. Scale bars: 200 µm (B,C); 50 µm (D); 100 µm (E).

Most studies using CF cultures rely on selective attachment to isolate the cell population, often without additional enrichment strategies (Garvin and Katwa, 2023). In our current study, we FACS-isolated RFP^+^ ventricular cardiac stromal cells from the Wt1^GFP/+^;Wt1Cre;ROSA26-tdRFP mouse model, excluding endothelial (RFP^+^, CD31^+^, CD45^−^) and hematopoietic (RFP^+^, CD31^−^, CD45^+^) cells, as well as cells with high GFP expression (i.e. epicardial cells among others), given that epicardial derivatives downregulate *Wt1* expression (Fig. 2A) (Feng et al., 2022; Pérez-Pomares et al., 2002; Velecela et al., 2019). The sorted RFP^+^ cells were cultured on poly-L-lysine-coated plates, where they exhibited a flattened, irregular morphology with abundant cytoplasmic protrusions (Fig. 2B). Next, the RFP^+^ cells were immortalized using a viral vector expressing the SV40 small T and large T antigen [CMV-SV40T (Puro)], which is well known for its robust immortalization ability and conferring puromycin resistance (Tevethia and Ozer, 2001). Following puromycin selection, the immortalized cells exhibited a more-uniform morphology with abundant cytoplasmic protrusions (Fig. 2C). Immunofluorescence analysis confirmed uniform expression of vimentin, which although not fibroblast specific – is abundantly expressed in this cell type (Fig. 2D) (Tallquist and Molkentin, 2017). Interestingly, despite this uniform vimentin expression, we observed discrete patches of α-smooth muscle actin (α-SMA)^+^ cells (Fig. 2E). Confocal imaging further revealed a strong and consistent colocalization of α-SMA and phalloidin-labeled F-actin, indicating the presence of a subset of activated fibroblasts within the culture (Fig. S3).

These results validate the successful immortalization of FACS-sorted RFP^+^ cardiac stromal cells.

### Characterization of cardiac RFP^+^ immortalized cells derived from the Wt1^GFP/+^;Wt1Cre;R26-tdRFP mouse model

**Fig. 3.**
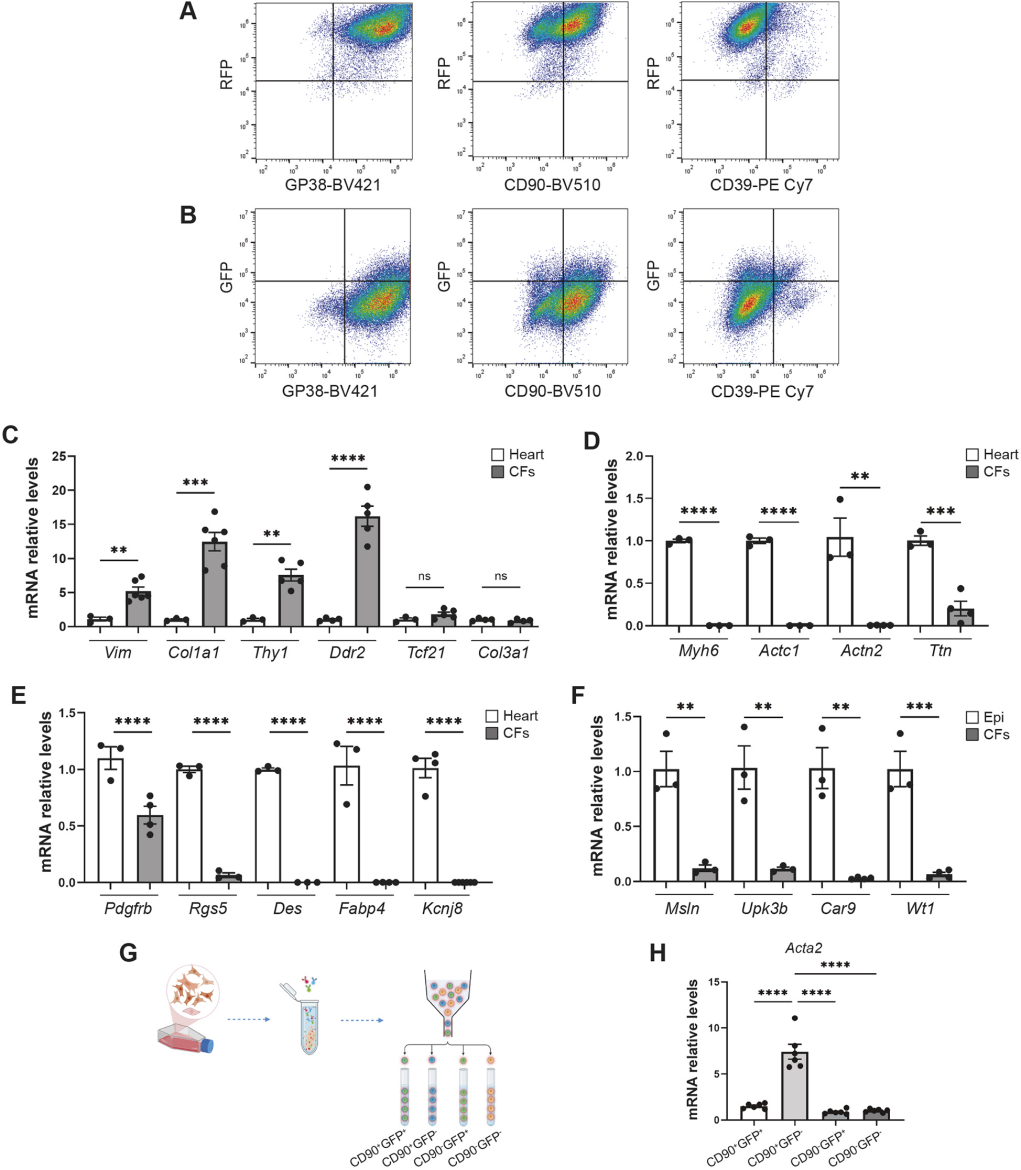
**Characterization of immortalized cardiac fibroblasts from Wt1^GFP/+^;Wt1Cre;ROSA26-tdRFP mouse model.** (A) Representative flow cytometry plots showing the identification of RFP^+^ cardiac fibroblast subsets. (B) Representative flow cytometry plots for identification of GFP^+^ subsets. (C-F) qRT-PCR analysis of the indicated genes in immortalized cardiac cells from Wt1^GFP/+^;Wt1Cre;ROSA26-tdRFP mice. Compared with heart ventricles or immortalized epicardial cells (Epi), immortalized fibroblasts exhibited abundant expression of CF marker genes (C) and almost undetectable expression of marker genes specific to cardiomyocytes (D), mural cells (smooth muscle cells and pericytes) (E) and epicardial cells (F). Data are presented as mean±s.e.m. (*n*=3-6); **P<0.01, ***P<0.001, ****P<0.0001, two-tailed Student’s *t*-test. (G) Schematic representation of the FACS-based isolation of four fibroblast subpopulations defined by CD90 and GFP expression. Created in BioRender by Martıńez-Estrada, O. M. (2025). https://BioRender.com/h3kmkr0. (H) qRT-PCR analysis of *Acta2* expression in these subsets revealed elevated *Acta2* levels in the CD90^+^GFP^−^ population. Data are presented as mean±s.e.m. (*n*=6); ****P<0.0001, one-way ANOVA followed by Tukey's post-hoc test.

Generating immortalized cells allows the study of CF signaling, proliferation and activation without much need to use animals. This can be achieved because the immortalized cells remain proliferative across multiple passages without showing signs of senescence, owing to SV40 expression in the immortalized cells (Fig. S4). Next, we aimed to characterize the RFP-immortalized cells in detail. Flow cytometry confirmed uniform RFP expression across all cells, with 15.49% expressing GFP (Fig. 3A, Table S5). When we included the antibodies used for *in vivo* analysis, flow cytometry revealed that 97.40% of RFP^+^ immortalized cells expressed GP38, 63.12% expressed CD90 and 5.87% expressed CD39 (Fig. 3A, Table S5). Consistent with *in vivo* observations, the proportion of GFP^+^ cells varied among subpopulations, i.e. being 10.14%, 11.61% and 29.98% among the RFP^+^GP38^+^, RFP^+^CD90^+^ and RFP^+^CD39^+^ subpopulations, respectively (Fig. 3B, Table S6).

Based on our cell isolation strategy and the predominance of CFs among epicardium-derived cells, we hypothesized that the immortalized cells are highly enriched in CFs. Thus, in addition to FACS analysis, we performed qRT-PCR, which confirmed that immortalized RFP^+^ cells from the Wt1^GFP/+;^Wt1Cre;R26-tdRFP mouse model express high levels of *Vim, Thy1*, *Col1a1*, *Col3a1, Tcf21* and *Ddr2* genes which are abundantly expressed in fibroblasts (Fig. 3C) (Tallquist and Molkentin, 2017).

To rule out the presence of other cell types in which Wt1Cre is active, such as mural cells (pericytes and smooth muscle cells) and cardiomyocytes (Forte et al., 2020; Wessels et al., 2012), we assessed the expression of genes enriched in these non-CF populations. Our analysis revealed that immortalized RFP^+^ cells showed almost undetectable expression of the cardiomyocyte marker genes (*Myh6*, *Actc1*, *Actn2*, *Ttn*) or mural cell marker genes (*Rgs5*, *Des*, *Fabp4*, *Kcnj8*, *Pdgfrb*) (Fig. 3D,E), providing strong evidence that this population is highly enriched in CFs (Forte et al., 2020; Skelly et al., 2018).

Next, we examined a selection of marker genes for epicardial cells, including *Upk3b*, *Msln, Car 9* and *Wt1* (Fig. 3F) (Du et al., 2023; Quijada et al., 2021; Velecela et al., 2019). qRT-PCR analysis showed that immortalized RFP^+^ cells expressed notably lower levels of these marker genes, confirming the effectiveness of our sorting strategy, i.e. one that excludes epicardial cells by removing cell populations with high GFP expression.

Having validated the CF identity of our culture and given that the vast majority of immortalized cells were GP38^+^CD39^−^, we FACS-sorted four distinct subpopulations based on CD90 and GFP expression: CD90^+^GFP^+^, CD90^+^GFP^−^, CD90^−^GFP^+^ and CD90^−^GFP^−^. We then assessed the potential contribution of each subpopulation to the α-SMA^+^ patches observed in culture. Interestingly, qRT-PCR analysis revealed that the CD90^+^GFP^−^ subpopulation expressed significantly higher levels of *Acta2* than the other subpopulations (Fig. 3G,H).

Collectively, these findings demonstrate that the RFP^+^ cells represent a heterogeneous population of CFs.

### TGFβ modulates the fibrotic response in RFP^+^ immortalized CFs

**Fig. 4.**
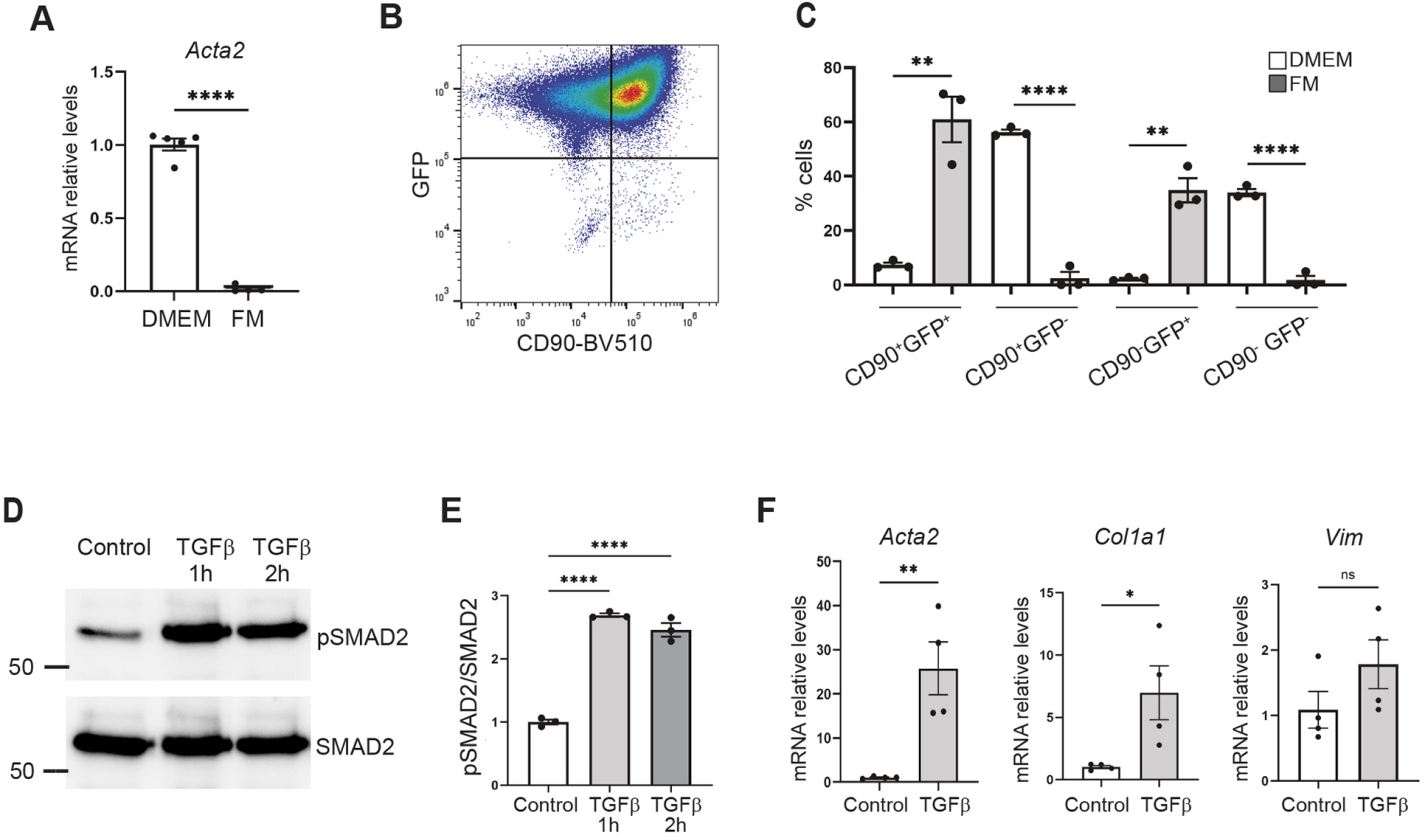
**TGFβ induces a profibrotic phenotype in immortalized RFP^+^ cardiac fibroblasts.** (A) qRT-PCR analysis of *Acta2* expression in immortalized RFP^+^ cardiac fibroblasts maintained in DMEM or fibroblast medium (FM). Data are presented as mean±s.e.m. (*n*=4-5); ****P<0.0001, two-tailed Student’s *t*-test. (B) Representative flow cytometry plots of immortalized RFP^+^ cardiac fibroblasts cultured in FM. (C) Quantification of CD90/GFP fibroblast subsets (CD90^+^GFP^+^, CD90^+^GFP^−^, CD90^−^GFP^+^, CD90^−^GFP^−^) in DMEM versus FM. Data are presented as mean±s.e.m. (*n*=3), **P<0.01, ****P<0.0001, two-tailed Student’s *t*-test. (D,E) Western blot and densitometric analysis of phosphorylated SMAD2 (pSMAD2) in immortalized cardiac fibroblasts treated with TGFβ, with total SMAD2 serving as the loading control. Data are presented as mean±s.e.m. (*n*=3); ****P<0.0001, one-way ANOVA followed by Tukey's post-hoc test. (F) qRT-PCR analysis of the indicated genes in immortalized cardiac fibroblasts cultured with TGFβ for 3 days. Data are presented as mean±s.e.m. (*n*=3), *P<0.05, **P<0.01, two-tailed Student’s *t*-test.

Increased expression of α-SMA is a widely used marker for identifying fibroblast activation (Garvin and Katwa, 2023). After observing α-SMA^+^ patches in our culture, we sought conditions able to prevent this activation. Commercial fibroblast-specific media reduce serum concentration, which helps prevent spontaneous activation. As expected, immortalized CFs cultured in this medium exhibited lower *Acta2* expression than those cultured in DMEM (Fig. 4A). Interestingly, this reduction in *Acta2* expression was accompanied by an increase in the CD90^+^GFP^+^ and CD90^−^GFP^+^ populations, along with a decrease in the CD90^+^GFP^−^ and CD90^−^GFP^−^ populations (Fig. 4B,C).

After successfully controlling the activation of our immortalized cell culture, our next objective was to investigate the effect of transforming growth factor beta (TGFβ), a central regulator of tissue fibrosis, and to assess whether our model could serve as an ideal system for studying cardiac fibrosis *in vitro* (Deng et al., 2024). Western blot analysis revealed a robust increase in the levels of phosphorylated SMAD2 (pSMAD2) in response to TGFβ, confirming the activation of the TGFβ-SMAD signaling cascade (Fig. 4D,E). To further characterize the fibrotic response of immortalized CFs to TGFβ, we examined the expression of two key fibrosis marker genes, *Acta2* and *Col1a1*. qRT-PCR analysis demonstrated significant upregulation of *Acta2* and *Col1a1* expression in TGFβ-treated CFs, highlighting the induction of a fibrotic phenotype. In contrast, expression of the pan-fibroblast marker gene *Vim* remained unchanged, suggesting that the observed changes are specific to a fibrotic phenotype (Fig. 4F).

Collectively, these findings demonstrate that our immortalized CFs constitute a robust and reliable model for studying stimulus-induced fibrotic activation in CFs.

## DISCUSSION

We developed a novel mouse model that enables distinct cardiac stromal cell populations, including resident fibroblasts, to be isolated based on WT1-reporter expression. The Wt1^GFP/+^;Wt1Cre;ROSA26-tdRFP model facilitates both the lineage tracing of cells labeled with Wt1Cre (RFP^+^) and the identification of cells actively expressing WT1 (GFP^+^). We generated an immortalized population enriched in different CF subtypes that retained RFP expression and expressed several marker genes that are highly enriched in fibroblasts, such as *Vim, Col1a1, Ddr2* and *Thy1*. The integrated GFP reporter enabled the isolation and functional analysis of distinct subpopulations within the Wt1Cre lineage.

During cardiac development, a subset of epicardial cells undergoes epithelial-to-mesenchymal transition, giving rise to EPDCs. These cells invade the subepicardial matrix before migrating into the myocardium, where they differentiate into various cardiac lineages, including most CFs (Gittenberger-de Groot et al., 1998). During early postnatal development, CFs undergo significant expansion via cell proliferation, which dramatically decreases after postnatal day (P)7 (Ieda et al., 2009; Wu et al., 2020). While several mechanisms that regulate the initial formation and differentiation of EPDCs have been elucidated, the molecular pathways controlling CF proliferation and terminal differentiation remain largely unknown (Tallquist, 2020). Given these limitations, the immortalized CFs generated in this study provide a valuable platform for screening candidate pathways involved in fibroblast function, serving as a complementary tool to *in vivo* studies.

The utility of this model also extends beyond developmental studies, because the adult mammalian heart has a limited capacity for regeneration. Healing typically occurs through scar formation, in which resident fibroblasts of epicardial origin play a central role (Tallquist, 2020). Although this response is essential for maintaining structural integrity and preventing ventricular rupture following injury, excessive or dysregulated activation can result in adverse cardiac remodeling, reduced contractile function and, ultimately, heart failure (Moore-Morris and Evans, 2023; Quijada et al., 2019). Notably, gene expression profiling has revealed that proliferating fibroblasts following myocardial infarction (MI) closely resemble neonatal fibroblasts underscoring the relevance of our model (Kretzschmar et al., 2018). Understanding the regulatory mechanisms that govern the expansion and differentiation of CFs into specific subpopulations could help develop therapeutic strategies that mitigate the effects of pathological fibrosis, while preserving essential wound healing processes.

WT1 is a transcription factor essential for development and tissue homeostasis (Hastie, 2017). Recent studies have highlighted its profibrotic role in lung fibrosis; however, WT1 deletion in the liver has also been shown to exacerbate fibrogenesis following injury (Kendall et al., 2019; Sontake et al., 2018). In the heart, studies using a zebrafish model of myocardial injury have shown that *wt1a:GFP^+^* cells upregulate a fibrotic gene program in response to damage (Sánchez-Iranzo et al., 2018). After analyzing different scRNA-seq datasets in mammals, WT1 has been classified as an early transcriptional regulator following myocardial infarction (Patrick et al., 2024). Despite these findings, the contribution of Wt1-expressing fibroblasts to the early stages of fibroblast activation and scar tissue formation after myocardial infarction remains unclear. Interestingly, CD90^+^GFP^−^ cells exhibited higher *Acta2* expression than CD90^+^GFP^+^ cells. Based on this, we hypothesize that, similar to TCF21, WT1 functions as a repressor of fibroblast activation and acquisition of a myofibroblast phenotype (Johansen et al., 2025). We propose that a gain-of-function approach may serve as an effective means to investigate WT1 function in this cell type both during development and following MI.

Currently, no effective pharmacological treatment for cardiac fibrosis exists, posing a major challenge in cardiovascular medicine. Our immortalized cells represent the first model of epicardial-origin CFs, offering a unique and valuable platform for studying the molecular mechanisms underlying cardiac fibrosis. Given their robustness and relevance, our model could prove highly effective for high-throughput drug screening, aiding in the identification of novel therapeutic agents that may reverse or alleviate cardiac fibrosis. Furthermore, the ability to genetically modify these cells facilitates the development of reporter-specific models, providing deeper insights into the cellular and molecular processes driving fibrosis in the heart and paving the way for more targeted, effective treatments.

In summary, we have developed a versatile model for studying CFs of epicardial origin. By adjusting the culture conditions, specific fibroblast subpopulations can be selectively promoted to enhance the flexibility of the model. The choice of conditions should be guided by the physiologically relevant context and desired subpopulation, which will allow not only screening for GFP expression but also for identifying treatment combinations that can modulate the proportions of different cell subpopulations. In turn, this can provide deeper insights into the functional roles of these cells.

## MATERIALS AND METHODS

### Animal models

The Wt1^GFP^ (Wt1^tm1Nhsn^) (MGI:3721102), ROSA26-tdRFP [Gt(ROSA)26Sor^tm1Hjf^] (MGI:3696099) and Wt1Cre [Tg(Wt1-cre)#Jbeb] (MGI:5308608) mice have been described previously (Hosen et al., 2007; Luche et al., 2007; Wessels et al., 2012). To generate Wt1Cre;ROSA26-tdRFP mice, male Wt1Cre mice were mated with ROSA26dtRFP females. To generate Wt1^GFP/+^;Wt1Cre;ROSA26-tdRFP mice, Wt1Cre;ROSA26-tdRFP males were mated with Wt1^GFP/+^ females. The primers used for genotyping are listed in Table S7.

All animal experiments were carried out in accordance with the regulations of the Animal Experimentation Ethics Committee (CEEA) of the University of Barcelona (ID C121CG2V6), thereby complying with current Spanish and European legislation.

### Flow cytometry analysis

Flow cytometry analyses were performed on freshly isolated cells from the cardiac ventricles of mice or on immortalized cells. For the *in vivo* analyses, early P2 heart ventricles from Wt1^GFP/+^;Wt1Cre;ROSA26-tdRFP mice and littermate negative controls were digested in 1 mg/ml collagenase type I solution (Worthington, 9001-12-1) for 30 min at 37°C, using a shaking block set to 1000 rpm (Eppendorf Thermomixer Compact). Collagenase activity was stopped by washing the cells with Dulbecco's modified Eagle’s medium (DMEM) (Gibco, 11960044) containing 10% fetal bovine serum (FBS; Capricorn Scientific, FBS-12B). Subsequently, the cells were then pelleted by centrifugation at 300 ***g*** for 5 min and filtered through a 40-μm cell strainer (Corning, 352340) to eliminate cardiomyocytes, before being resuspended in Hanks' Balanced Salt Solution (HBSS Ca^2+^, Mg^2+^; Gibco, 14025092) containing 2% FBS (FACs buffer) (Ramiro-Pareta et al., 2023; Torres-Cano et al., 2022).

For antibody staining, freshly isolated or immortalized cells were incubated with primary monoclonal or isotype control antibodies at 4°C for 30 min (Table S8) (Ramiro-Pareta et al., 2023). Samples were washed twice and resuspended in FACs buffer. Flow cytometry analysis was carried out using a Cytek^®^ Aurora 4 L (Cytek) and data were analyzed using FlowJo™ software (v10.10). Cell gating of the corresponding fluorescent protein was carried out using samples negative for the fluorescent transgenic protein and isotype control antibodies.

### Immortalization of RFP^+^ cardiac cells

RFP^+^ cells were isolated by FACS (FACSAria™ Fusion I, BD) from the enzymatically digested P2 hearts of Wt1^GFP/+^;Wt1Cre;ROSA26-tdRFP mice, excluding endothelial (RFP^+^, CD31^+^, CD45^−^), hematopoietic (RFP^+^, CD31^−^, CD45^+^) and epicardial (RFP^+^, GFP^++^) cell populations. The sorted RFP^+^ cells were subsequently cultured on 24-well plates coated with poly-L-lysine (Sigma, P7886) in completed cell culture medium (DMEM high-glucose; Gibco, 11960044) supplemented with L-glutamine (Corning, 25-005-CV), 100 µg/ml streptomycin, 100 U/ml penicillin (Gibco, 15140122) and 10% FBS (Capricorn Scientific, FBS-12B) until reaching ∼70% confluence.

To induce immortalization, cells were incubated overnight at 37°C with 1.2 µl of SV40 viral vector supernatant [CMV-SV40T (Puro) Lentifect™, GeneCopoeia, LP721-025]. After 24 h, the supernatant was removed and fresh culture medium was added. Cells were then cultured for an additional 21 days in presence of puromycin (Sigma, P8833). Non-transduced cells served as controls for the immortalization process. Once confluence was achieved, cells were trypsinized and designated as passage 1.

The immortalized cells, negative for bacterial and mycoplasma contamination, were maintained in complete DMEM culture medium and passaged every 4 to 5 days. Successful immortalization was confirmed by qPCR analysis of SV40 gene expression, with amplicons visualized on a 2% agarose gel (Panreac, A8963,0500). HEK293T and human umbilical vein endothelial cells were used as positive and negative controls, respectively.

### Fibroblast medium and TGFβ treatment conditions

Immortalized cells were maintained in complete DMEM culture medium and passaged every 4 to 5 days upon reaching 70% confluency. To assess the impact of fibroblast medium on cellular phenotype and stability of marker gene expression, cells were cultured in a commercially available fibroblast medium (Promocell, C-23025) for three passages, followed by subsequent analyses. Cells between passages 6 and 16 were used for all experiments.

To investigate the effect of TGFβ stimulation, cells were cultured at a density of 10,000 cells/cm² in fibroblast growth medium for 24 h, followed by overnight incubation in Opti-MEM medium. TGFβ (10 ng/ml) was then applied for 1, 2 or 72 h, after which samples were collected for marker gene expression analysis by western blot or qPCR respectively.

### Isolation of RNA and real-time PCR

Total RNA from RFP^+^ cell cultures, FACS-sorted subpopulations and immortalized epicardial cells (Guadix et al., 2011) was isolated using the PureLink RNA Mini Kit (Invitrogen, 12183025). RNA from heart ventricles was isolated using the RNeasy Micro Kit (Qiagen, 74004), following the manufacturer's instructions. The purified RNA was reverse transcribed into cDNA using UltraScript^®^ Reverse Transcriptase (PCR Biosystems, PB30.11-10). Relative gene expression levels were quantified using SYBR Master Mix (Promega, A6002); real-time PCR was performed on 96-well QuantStudio 3 plates (Applied Biosystems) using Connect analysis software (Thermo Fisher Scientific). The primers used are listed in Table S9.

### Immunofluorescence

Immortalized cells were seeded on 12 mm glass coverslips (6000 cells/cm²) and cultured for 48 h in complete DMEM culture medium or fibroblast growth medium. Cells were then fixed with 4% paraformaldehyde (Sigma, 158127) for 15 min, washed with phosphate buffered saline (PBS), permeabilized with 0.1% Triton X-100 (Sigma, 9036-19-5) for 5 min, washed again with PBS and blocked with PBS supplemented with 1% bovine serum albumin (Sigma, 810664) for 1 h at room temperature. After blocking, cells were incubated overnight at 4°C with primary antibodies against vimentin and α-SMA along with phalloidin, to stain filamentous actin (Table S10). Then, the cells were incubated with fluorochrome-conjugated secondary antibodies (Table S10) for 1 h at 37°C. Cell nuclei were then stained with DAPI (Sigma, D9542). Immunofluorescence images were obtained using a confocal microscope (ZEISS LSM900).

### Image processing and colocalization metrics (α-SMA)

Quantitative colocalization between α-SMA and phalloidin signals was assessed using the Coloc2 plugin in ImageJ/FIJI. Prior to analysis, background signal was removed using Subtract Background (rolling ball radius: 50 pixels). Thresholded Manders' coefficients (M1 and M2) were automatically calculated to determine the degree of fluorescence overlap between the two channels across ten images, with values ≥0.90 interpreted as indicative of relevant colocalization.

### Western blotting

For western blot analyses, control and cells treated with TGFβ (10 ng/ml for 1 and 2 h) were lysed using SDS buffer (1.5 M Tris pH 6.8, 15% glycerol, 3% SDS, 7.5% β-mercaptoethanol and 0.0375% Bromophenol Blue). Lysates were separated by SDS-PAGE gel and transferred to PVDF membranes for immunoblotting. Membranes were blocked for 1 h at room temperature in Tris-buffered saline containing 0.1% Tween-20 (TBST) and 5% bovine serum albumin (BSA) and immunoblots were incubated overnight at 4°C with antibodies against SMAD2 and phosphorylated SMAD2 (pSMAD2). Signal detection was performed using HRP-conjugated IgG secondary antibodies (Table S11). Blot intensities were quantified using ImageJ software and one-way ANOVA followed by Tukey's post hoc test was used to examine statistical significance.

### Quantification and statistical analysis

Data are presented as mean±s.e.m. Statistical significance between two groups was determined using an unpaired, two-tailed Student's *t*-test. For comparisons among multiple groups, one-way ANOVA followed by Tukey's post hoc test was applied. Statistical analyses were performed using GraphPad Prism version 10 (GraphPad Software).

## Supplementary Material

10.1242/dmm.052601_sup1 Supplementary information
