# Supplementary material for: An innovative in vitro model for studying the biology of cardiac fibroblasts originating from the epicardium
Source: Dis Model Mech. 2026 Apr 2;19(3):dmm052601. doi: 10.1242/dmm.052601 (PMC13072145; doi:10.1242/dmm.052601)
Supplement: Supplementary information [file dmm-19-052601-s1.pdf]

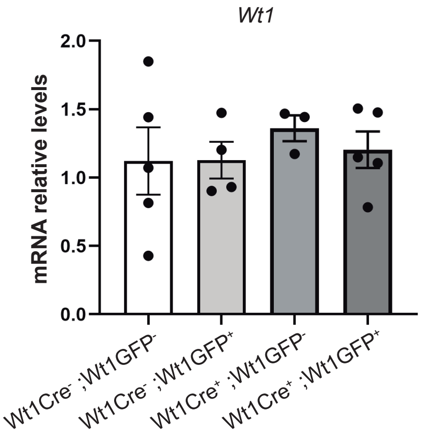

Fig. S1. Comparable *Wt1* transcript levels across genotypes from the Wt1Cre;ROSA26-tdRFP × Wt1$^{GFP/+}$ cross. qRT–PCR of *Wt1* expression in heart ventricles of the indicated genotypes at P0. Data are presented as mean ± s.e.m. (n = 3–5), one-way ANOVA followed by Tukey's post-hoc test.

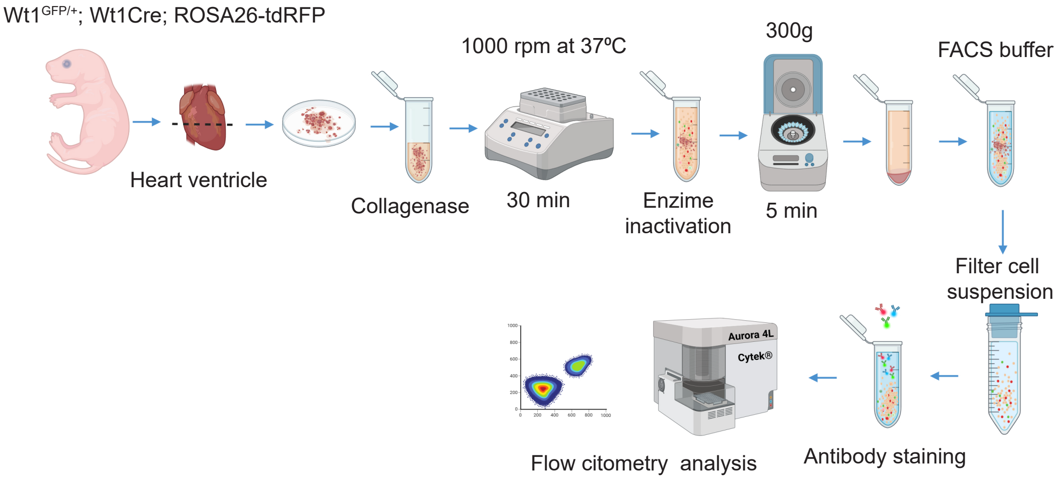

Fig. S2. Experimental workflow for cardiac ventricular digestion and flow cytometry analysis. Overview of the protocol of the enzymatic digestion and flow cytometry analysis of heart ventricles from Wt1$^{GFP/+}$;Wt1Cre;ROSA26-tdRFP mice.Created in BioRender by Martıńez-Estrada, O. M. (2025). https://BioRender.com/7ru99s1.

Disease Models & Mechanisms • Supplementary information

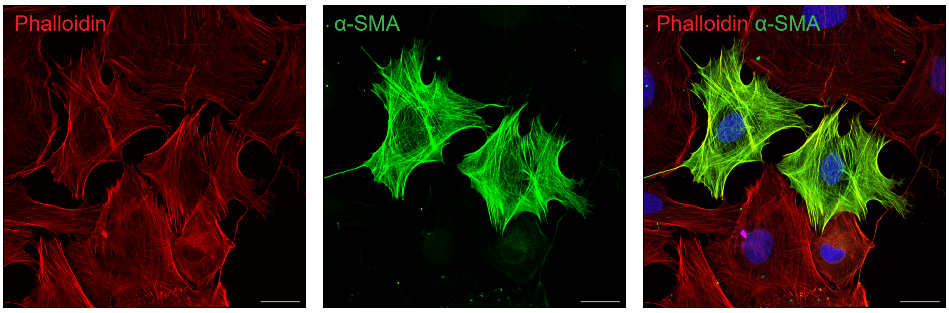

Fig. S3. Colocalization of α-SMA and phalloidin in immortalized RFP$^+$ cardiac cells. Representative high magnification images of α-SMA (green) and phalloidin (red), confirming robust colocalization (Manders' coefficients >0.90). Scale bar: 25 μm.

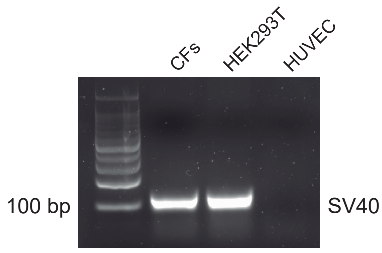

Fig. S4. SV40 expression in immortalized RFP$^+$ cardiac cells. Amplified qPCR product for the SV40 gene was loaded onto a 2% agarose gel, showing SV40 expression in cardiac fibroblasts (CFs) and in HEK293T cells (positive control), with no detectable expression in HUVEC cells (negative control).

Disease Models & Mechanisms • Supplementary information

Table S1. Percentage of RFP-positive cells relative to the total number of cells, determined by flow cytometry. Data are mean ± SD (n=4).

	**% RFP/total cells**
**r1**	16.65
**r2**	12.25
**r3**	12.85
**r4**	13.25
**Mean**	**13.75**
**SD**	**1.98**

Table S2. Percentage of cells expressing the indicated markers within the RFP-positive cell population, determined by flow cytometry. Data are mean ± SD (n=4).

	**RFP+ cells**					
	**GFP**	**GP38**	**CD90**	**CD39**	**CD31**	**CD45**
**r1**	20.5	79.90	19.4	30.50	22.60	24.50
**r2**	24.1	69.50	19.9	30.90	24.70	24.50
**r3**	23.8	69.20	15.8	31.30	24.50	25.40
**r4**	12.10	74.40	17	23.40	31.50	34.60
**Mean**	**20.13**	**73.25**	**18.03**	**29.03**	**25.83**	**27.25**
**SD**	**5.59**	**5.03**	**1.95**	**3.76**	**3.90**	**4.92**

Table S3. Percentage of GFP cells within the RFP+ CD31+ and RFP+ CD45+ cell population, determined by flow cytometry. Data are mean ± SD (n=4).

	**% GFP+ cells**	
	**RFP+/CD31+**	**RFP+/CD45+**
**r1**	73.38	64.69
**r2**	79.96	76.50
**r3**	76.45	69.65
**r4**	75.58	67.59
**Mean**	**76.34**	**69.61**
**SD**	**2.74**	**5.03**

Disease Models & Mechanisms • Supplementary information

Table S4. Percentage of cells expressing the indicated markers within the RFP-positive cell population, determined by flow cytometry. Data are mean ± SD (n=4).

	RFP+/GP38+/			RFP+/CD90+/			RFP+/CD39+/		
	**GFP**	**CD90**	**CD39**	**GFP**	**GP38**	**CD39**	**GFP**	**GP38**	**CD90**
**r1**	29.69	22.92	27.87	38.94	85.86	34.43	70.97	77.76	21.82
**r2**	37.09	23.18	24.90	46.52	76.14	45.66	78.65	57.96	8.68
**r3**	30.81	18.69	24.54	32.06	75.82	28.65	77.70	58.93	9.44
**r4**	18.06	20.23	16.95	28.33	80.06	38.28	56.84	57.67	18.73
**Mean**	**28.91**	**21.25**	**23.56**	**36.46**	**79.47**	**36.76**	**71.04**	**63.08**	**14.67**
**SD**	**7.93**	**2.17**	**4.65**	**8.02**	**4.68**	**7.13**	**10.07**	**9.80**	**6.61**

Table S5. Percentage of immortalized cells expressing the indicated markers within the RFP-positive cell population, determined by flow cytometry. Data are mean ± SD (n=3).

	Immortalized cells RFP+			
	**GFP**	**GP38**	**CD90**	**CD39**
**r1**	18.62	98.04	64.44	7.79
**r2**	14.87	97.33	63.95	5.67
**r3**	12.99	96.84	60.97	4.14
**Mean**	**15.49**	**97.40**	**63.12**	**5.87**
**SD**	**2.87**	**0.60**	**1.88**	**1.83**

Table S6. Percentage of immortalized cells expressing the indicated markers within the RFP-positive cell population, determined by flow cytometry. Data are mean ± SD (n=3).

	RFP+/GFP+/			RFP+/GP38+/			RFP+/CD90+/			RFP+/CD39+/		
	**GP38**	**CD90**	**CD39**	**GFP**	**CD90**	**CD39**	**GFP**	**GP38**	**CD39**	**GFP**	**GP38**	**CD90**
**r1**	99.83	78.25	30.27	12.07	66.05	6.63	14.06	99.44	10.96	42.82	89.44	82.18
**r2**	99.12	70.50	17.16	9.75	66.01	4.69	10.23	99.01	8.14	26.83	91.19	82.60
**r3**	99.42	79.51	12.27	8.59	63.25	4.06	10.52	99.00	6.26	20.28	88.58	74.09
**Mean**	**99.46**	**76.09**	**19.90**	**10.14**	**65.11**	**5.13**	**11.61**	**99.15**	**8.45**	**29.98**	**89.73**	**79.62**
**SD**	**0.36**	**4.88**	**9.31**	**1.77**	**1.61**	**1.34**	**2.13**	**0.25**	**2.36**	**11.59**	**1.33**	**4.80**

Table S7. List of PCR primers used for genotyping

Gene	Primers (5’ - … - 3’)
Cre	Cre F: GCATTACCGGTCGATGCAACGAGTGATGAG<br>Cre R: GAGTGAACGAACCTGGTCGAAATCAGTGCG<br>Fabpi200 F: TGGACAGGACTGGACCTCTGCTTTCCTAGA<br>Fabpi200 R: TAGAGCTTTGCCACATCACAGGTCATTCAG
$Wt1^{GFP}$	Wt1-GFP F: GCCTGAAGAACGAGATCAGC<br>Wt1-GFP F2: AGCCTGAAGCTGCTCACATCC<br>Wt1-GFP R: GGCAGCTTGAATTCCTCTCA
RFP	R26g2 F: TGTTATCAGTAAGGGAGCT<br>R26g2 R mut: AAGACCGCGAAGAGTTTGT<br>R26g2 R wt: CACACCAGGTTAGCCTTTA

Table S8. List of antibodies used for flow cytometry analysis

Antibody	Manufacturer	Cat. Number	Dilution
APC Rat anti-mouse CD31 (Clone: MEC 13.3)	BD Pharmigen™	561815	1:1000
PE-Cy™7 Rat anti-mouse CD45 (Clone: 30-F11)	BD Pharmigen™	552848	1:1000
Brilliant Violet 510™ Rat anti-mouse CD90.2 (Thy-1.2)	BioLegend	140319	2 µl per million cells in 100 µl
Brilliant Violet 421™ Syrian Hamster anti-mouse Podoplanin	BioLegend	127423	≤ 0.25 µg per million cells in 100 µl
PE-Cy™7 Rat anti-mouse CD39	BioLegend	143805	≤ 1.0 µg per million cells in 100 µl

Disease Models & Mechanisms • Supplementary information

Table S9. qRT-PCR primer list.

Gene	Primers (5’ - … - 3’)
Vim	F: CCAACCTTTTCTTCCCTGAA<br>R: TGAGTGGGTGTCAACCAGAG
Col1a1	F: CATGTTCAGCTTTGTGGACCT<br>R: GCAGCTGACTTCAGGGATGT
Col3a1	F: CCTGGCTCAAATGGCTCAC<br>R: CAGGACTGCCGTTATTCCCG
Thy1	F: GGTGGCAGAAGAAGACAAGG<br>R: CCTTCCTGCACGGACTTAGA
Tcf21	F: TGATTAACTTCTGCCATGAATGA<br>R: AAAAGATACACATTGATAGGCTCTTCT
DDR2	F: TCATCCTGTGGAGGCAGTTCTG<br>R: CTGTTCACTTGGTGATGAGGAGC
Desmin	F: GCGTGACCAACCTGATAGACG<br>R: GTTGGATTTCCTCCTGTAGTT
Rgs5	F: AAGTTGGGAATTCTCCTCCAG<br>R: TTCCTCACTGAATTCAGACTTC
Fabp4	F: GGATGGAAAGTCGACCACAA<br>R: TGGAAGTCACGCCTTTCATA
Kcnj8	F. CACAAGAACATCCGAGAGC<br>R: GGGCATTCCTCAGTCATCAT
PDGFrβ	F: TGCAGAGACCTCAAAAGGTG<br>R: CCTGATCTTCCTCCCAGAAA
Myh6	F: TGCTCAGAGCTCAAGAAGGAT<br>R: CCCAGCCATCTCCTCTGTTA
Actc1	F: CCGATCGTATGCAAAAGGAA<br>R: CTGGAAGGTGGACAGAGAGG
Actn2	F: CTCGGAGCTCCATCCAGA<br>R: ACTGCTTCAGCTGGTTCATCT
Ttn	F: CCGGTGGAAGCTGCTATATT<br>R: CCGAGGTTTTCACTGCGTA
Msln	F: CTGCAGACCCAGACTACAA<br>R: GAGGCCTGTGGGGAGACT
Upk3b	F: CCTGTCCAGATGTGGGATCT<br>R: TCCGAGGATAGTTTGAGAGCA
Car9	F: GCCCAGAAGAAAACAGTGCT<br>R: CCAAACCTGGGATCTCAATC
Wt1	F: TTCAAGGACTGCGAGAGAAG<br>R: GGGAAAACTTTCGCTGACAA
Acta2	BioRad #10041595
SV40	F: GTCTTCTACCTTTCTCTTCTTT<br>R: GGAGCAGTGGTGGAA

Disease Models & Mechanisms • Supplementary information

Table S10. List of primary and secondary antibodies used for immunofluorescence.

Antibody	Manufacturer	Cat. Number	Species	Dilution
Anti-Vimentin	Abcam	ab8978	Mouse	1:100
Anti-αSMA	Cell Signalling	19245S	Rabbit	1:100
Phalloidin-TRITC	Sigma	P1951		1:200
Anti-mouse AlexaFluor 546	Invitrogen	A11003	Goat	1:400
Anti-rabbit Alexa Fluor 633	Invitrogen	A21070	Goat	1:400

Table S11. List of antibodies used for Western Blot analysis.

Antibody	Manufacturer	Cat. Number	Species	Dilution
p-SMAD2	Cell Signalling	3108	Rabbit	1:1000
SMAD2	Cell Signalling	3103	Mouse	1:1000
Anti-Rabbit IgG HRP conjugate	BioRad	1706515	Goat	1:3000
Anti-Mouse IgG HRP conjugate	BioRad	1706516	Goat	1:3000

Disease Models & Mechanisms • Supplementary information
